# CERES-Maize model for simulating genotype-by-environment interaction of maize and its stability in the dry and wet savannas of Nigeria

**DOI:** 10.1016/j.fcr.2020.107826

**Published:** 2020-08-15

**Authors:** A.A Adnan, J. Diels, J.M. Jibrin, A.Y Kamara, A.S Shaibu, P Craufurd, Abebe Menkir

**Affiliations:** a Department of Agronomy, Bayero University Kano, 70001, Kano, Nigeria; b Department of Earth and Environmental Sciences, Division of Soil and Water Management, KU Leuven, Celestijnenlaan 200E, 3001 Leuven, Belgium; c Centre for Dryland Agriculture (CDA), Bayero University Kano, 70001, Kano, Nigeria; d International Institute of Tropical Agriculture, Ibadan, Nigeria. c/o IITA Ltd, Carolyn House, 26 Dingwall Road, Croydon CR9 3 EE, United Kingdom; e International Maize and Wheat Improvement Center (CIMMYT) World Agroforestry Centre (ICRAF) House United Nations Avenue, Gigiri P.O. Box 1041–00621, Nairobi, Kenya

**Keywords:** CERES-Maize model, Genotype by environment interaction, Stability analysis

## Abstract

When properly calibrated and evaluated, dynamic crop simulation models can provide insights into the different components of genotype by environment interactions (GEIs). Modelled outputs could be used to complement data from multi-environment trials. Field experiments were conducted in the rainy and dry seasons of 2015 and 2016 across four locations in maize growing regions of Northern Nigeria using 16 maize varieties planted under near-optimal conditions of moisture and soil nitrogen. The CERES-Maize model was calibrated using data from three locations and two seasons (rainy and dry) and evaluated using data from one location and two seasons all in 2015. Observed data from the four locations and two seasons in 2016 was used to create eight different environments. Two profile pits were dug in each location and were used separately in the simulations for each environment to provide replicated data for stability analysis in a combined ANOVA. The effects of the environment, genotype and GEI were highly significant (p = 0.001) for both observed and simulated grain yields. The environment explained 67 % and 64 % of the variations in observed and simulated grain yields respectively. The variance component of GEI (13 % for observed and 15 % for simulated) were lower but still considerable when compared to that of genotypes (19 % for observed and 21 % for simulated). From the stability analysis of the observed and simulated grain yields using six different stability models, three models (ASV, Ecovalence, and Sigma) ranked Ife Hybrid as the most stable variety. The slope of the regression (bi) model ranked Sammaz 11 as the most stable variety, while the Shukla model ranked Sammaz 28 as the most stable variety. Long-term seasonal analysis with the CERES-Maize model revealed that early and intermediate maturing varieties produce high yields in both wet and dry savannas, early and extra-early varieties produce high yields only in the dry savannas, while late maturing varieties produce high yields only in the wet savannas. When properly calibrated and evaluated, the CERES-Maize model can be used to generate data for GEI and stability studies of maize genotype in the absence of observed field data.

## Highlights

• Genotype by Environment Interaction (GEI) makes it difficult for breeders and growers to select stable, high yielding varieties across different environments thereby reducing the effectiveness of the selection process.
• Determining the magnitude of GEI and the stability of varieties can be challenging, as such, crop models can be employed to complement this process.
• Dynamic models that can simulate the response of growth and development of crops to varying abiotic environmental factors have the potential to explain yield differences due to temporal and spatial variability.
• Crop Simulation Models were used to complement multi environment trials (METs) with a view to enhancing selection of high yielding varieties across multiple locations.
• The model simulations matched actual observations and produced similar ranking, indicating that properly calibrated and evaluated CERES-Maize model can complement METs.

## 1 Introduction

In the savannas of Nigeria, including the semi-arid Sudan savanna zone, maize production has increased greatly in the past three decades (IITA, 2017). Maize has evolved from a backyard crop, produced mainly by women and children, to a major commercial crop providing food, animal feed, and industrial raw materials (Badu-Apraku and Fakorede, 2017). The total annual national production in Nigeria increased from 1.06 M tons in 1976, to about 11.6 M tons in 2016 (FAO, 2018). The annual increase in production output is majorly attributed to the increase in the production area and not the much-needed intensification. Average yields per hectare have been below 2 Mg ha^−1^ since 1965 except for 2009 where a national average of 2.2 Mg ha^-1^ was reported (FAO, 2018). The national average figures are quite low due to a large number of farmers having yields below 1.4 Mg ha^−1^, but yields of >7 Mg ha^−1^ have been reported in research stations and across best-farmer fields (IITA, 2017). The high yields in research stations and best-farmer fields are mainly due to the selection of appropriate maturity groups, high yielding varieties and adoption of best agronomic practices (Kamara et al., 2009).

Over the years, many maize varieties of contrasting characteristics that are adapted to the different regions of Nigeria have been developed by the International Institute of Tropical Agriculture (IITA) and partners (Badu-Apraku et al., 2011). These varieties have high yield potentials and additional characteristics such as tolerance to drought and low nitrogen as well as biotic stresses including the parasitic weed, *Striga hermonthica* (Ifie et al., 2015). The selection of such varieties by smallholder farmers is largely dependent on grain yields because they are always the traits of economic relevance. Changes in the relative grain yield output and other traits of genotypes in different production environments are usually observed via a phenomenon called genotype by environment interaction (GEI) (Badu-Apraku et al., 2003). For a variety to be selected and utilized in a large group of environments, evaluating the stability of performance and range of adaptation has become increasingly important. The GEI makes it difficult for breeders and growers to select high yielding varieties that are stable across different environments thereby reducing the effectiveness of the selection process (Yan and Hunt, 2010). Furthermore, determining the magnitude of GEI and the stability of varieties can be challenging, as such, crop models can be employed to complement this process.

Dynamic models that can simulate the response of growth and development of crops to varying abiotic environmental factors such as temperature, solar radiation, and daylength have the potential to explain yield differences due to temporal and spatial variability (Sadras et al., 2003). These models can also be used to explain yield variability for different varieties across varying environments and management conditions thereby quantifying the GEI (Bustos-Korts et al., 2016). The models become more useful when they integrate a plant-soil-weather-management continuum which gives them the potential to provide a site and variety-specific agronomic recommendations for practices like optimum sowing dates/density and appropriate fertilization requirements (Magaia et al., 2017). Several efforts by researchers to integrate breeding and crop modelling have been well documented (Chapman, 2008; Chapman et al., 2002b). Crop growth models used in plant breeding are centered on explaining resource capture, utilization and allocation among plant organs (Cooper et al., 2009; Hammer et al., 2006). They have also been used to characterize growing environments (Chapman et al., 2000; Seyoum et al., 2017), to predict the influence of trait variation on yield within the context of genotype by environment by management interaction (Löffler et al., 2005; Seyoum et al., 2018), for evaluation of breeding strategies (Chapman et al., 2003) and to assess hybrid performance (Cooper et al., 2014). However, very few studies have reported a comparison between simulated and observed values on GEI and stability analysis (Chapman, 2008). Several kinds of research have shown that models can be used to simulate GEI (Salmerόn et al., 2017; Mavromatis et al., 2002; van Eeuwijk et al., 2019; Chapman et al., 2002a; Seyoum et al., 2018). However, all the studies were done in controlled experiments under the close supervision of scientists. None of the studies were conducted in farmer fields under partial supervision of scientists. Additionally, none of the model based GEI studies were evaluated using different stability parameters especially for maize. In Africa, studies have been conducted using APSIM to characterize production environments and simulate GEI for maize, however, we could not find any available literature for comparisons between model-based and field observed GEI as well as the evaluation of the stability of varieties using different stability models.

The CERES-Maize model which was used in this study is a dynamic crop simulation model that estimates maize phenology, dry matter production/partitioning, and yield in daily time steps (Jones et al., 1986). Over the years, the use of the CERES-maize model in making management decisions has been increasing in Africa. For instance, it was used to; evaluate climate-sensitive farm management practices in the Northern Regions of Ghana (MacCarthy et al., 2017); identify appropriate sowing dates and nitrogen rates in Zambia (Chisanga et al., 2014); simulate nitrogen and phosphorus uptakes and soil moisture dynamics in West Africa (Amouzou et al., 2018); and recently used in Benin Republic to provide support decision making regarding fertilizer micro-dosing for maize production (Tovihoudji et al., 2019). In Nigeria, the model has been used for the determination of the nitrogen fertilization requirements and optimum planting dates of maize (Adnan et al., 2017a, 2017b). Considering the increased use of the model in Africa, there is need to test the capacity of the model in predicting GEI in field trials and breeders’ program. There is also a need to test the stability of the model simulated grain yields for different maize varieties across varying environments. Therefore, the objectives of the present research were to (i) evaluate the applicability of the CERES-Maize model in predicting genotype-by-environment interaction and (ii) compare the stability of observed and simulated grain yields of 16 maize varieties across diverse environments.

## 2 Materials and methods

### 2.1 Experimental conditions

Field experiments were conducted during the rainy and dry seasons of 2015 and 2016 across four locations in northern Nigeria (Table 1). The experimental locations include: Kano (BUK) (N11.516 E8.516 466 m asl), Dambatta (DBT) (N12.333 E8.517 442 m asl), Samaru (SMR) (N11.187 E7.147 702 m asl) and Lere (LER) (N10.52 E8.472 786 asl). In 2015, the trials in BUK were sown on 20th March and 14th July in BUK, 21st March and 15th July in DBT, 23rd March and 17th July in SMR and LER. In 2016 however, sowing was done for the two seasons on 16th March and 20th July in BUK, 19th March and 21st July in DBT, 22nd March and 22nd July in SMR and 17th March and 24th July in LER. The experiments were conducted near irrigation facilities to maintain optimum moisture by irrigating when the soil moisture is below field capacity for both the dry and rainy season trials. Moisture conditions were monitored using a Time Domain Reflectometry (TDR) Meter 6050 × 1 TRASE SYSTEM (Soil moisture Equipment Corporation). Mineral fertilizers were applied at the rate of 120N:60P_2_O_5_:60K_2_O kg ha^−1^); potassium (K) was applied in form of Muriate of Potash, phosphorus in the form of Single Super Phosphate, and Nitrogen was applied in the form of Urea. While all the P and K fertilizers were applied at sowing only half of the N fertilizer was applied at the time of sowing and the other half applied 21 days later. Also, poultry manure (approximately NPK 1.1:0.8:0.5) was added to the fields at the rate of 5 Mg ha^−1^ to maintain optimum nutrient status. The experimental fields were cleared, harrowed, ridged and thereafter sprayed with a pre-emergence herbicide, Primextra (Atrazine + Metolachlor) at the rate of 4lha^−1^ before planting. The maize was sown at an intra-row spacing of 0.25 m at two seeds per hole and later thinned to one plant giving a population of 53,333 plants ha^−1^. The experiments were laid out in a Randomized Complete Block Design (RCBD) with four replications. The gross plot consisted of six rows 0.75 m apart and 3 m long (plot area = 13.5 m^2^). The two innermost rows were used as the net plot for yield assessment and sampling purposes. A space of 0.5 m was used between plots and 1 m between replications.

**Table 1:** Description of environments in the study.

Environment	Environment Code	Season	Location	Soil Type
E1	DSBUK	Dry	Bayero University	Typic Kandiustalf
E2	DSDBT	Dry	Dambatta	Typic Kanhaplustalf
E3	DSLERE	Dry	Lere	Plinthic Kandihumult
E4	DSSMR	Dry	Samaru	Plinthic Haplustult
E5	RSBUK	Rainy	Bayero University	Typic Kandiustalf
E6	RSDBT	Rainy	Dambatta	Typic Kanhaplustalf
E7	RSLERE	Rainy	Lere	Plinthic Kandihumult
E8	RSSMR	Rainy	Samaru	Plinthic Haplustult

### 2.2 Plant data measurements

Detailed data for growth, phenology and yield were collected and used for model calibration and evaluation, while only grain yield data was used for GEI and stability analysis. Evaluation of crop development was done by observing the phenology of the different maize varieties and recording the length of time (days) it takes to attain each phenological phase. The measurements were then converted to growing degree days (GDD) using a base temperature of 8 °C and adopting the relationship:

(1) $$\;GDD=\sum_{i=1}^{n}\left(\frac{{Tmax}_{i}+{Tmin}_{i}}{2}\right)-Tbase$$

Where, GDD is the growing degree days, *Tmax_i_* is the maximum temperature for the ith day, *Tmin_i_* is the minimum temperature for the ith variety and *Tbase* is the base temperature for maize.

Ten plants were tagged from the center of each plot in each replication for phenological observations. The end of the juvenile stage (i.e. panicle initiation) was determined through destructive sampling and dissection of three plants, followed by observation of apical meristem to check for floral bud development at two days intervals starting from 14 days after emergence. The end of the juvenile stage was recorded when the male flower primordial was visible in 50 % of plants examined. Days to 50 % tasselling was recorded when tassels were observed on 50 % of the tagged plants. Physiological maturity observations were conducted as follows: kernels were removed from the base, middle and distal end of each sampled ear daily, starting when husks began to show signs of drying. Days to physiological maturity was recorded when 50 % of the kernels in each tagged ear had formed a black layer, indicating physiological maturity. Plant biomass was taken at four different stages: vegetative, anthesis, grain filling and physiological maturity. Five plants within a one-meter strip in a row were cut at the ground, leaves were separated from the stem, chopped and dried in the shade for three days. Both stems and leaves were oven-dried at 70 °C for 36–48 hours until the sample had attained constant weight. Yield and yield component measurements were taken at harvest maturity. Other variables measured included: the number of seeds per unit area (seed # m^−2^), dry seed weight (g m^−2^), dry cob weight (g m^−2^), dry husks weight (g m^−2^), grain yield (kg ha^-1^) and stover weight at harvest (kg ha^-1^). All yield and yield component measurements were done using procedures and formulae described by Ogoshi et al. (1999).

### 2.3 Initialization of soil and weather parameters

Two soil profile pits were dug in each location before planting, and soil samples were taken from each layer for detailed studies. The two profile pits used separately in the simulations for each environment to provide replicated data for stability analysis. The samples from each layer were analyzed for pH (in H_2_0), texture, moisture, bulk density, exchangeable potassium (K), organic matter, available phosphorus (Bray II), total nitrogen and CEC. The soil data tool (*SBuild*) of DSSAT was used to create the soil database which was used for general simulation purposes. The parameterization of SBuild for calibration and simulation experiments were done as previously reported (Adnan et al., 2019).

Daily weather data was collected from weather stations (Watchdog 2000 Series, Spectrum Technologies) adjacent to all experimental sites. All the weather stations were less than 2 km away from the experimental sites. The *Weatherman* utility in DSSAT was used to input the weather data to create the weather file used by the CERES- Maize model (Adnan et al., 2019).

For other simulation options, initial conditions were as reported for each year and location, the Priestly-Taylor/Ritchie method was selected for simulation of evapotranspiration while the Soil Conservation Service (SCS) method was selected for simulation of infiltration. Photosynthesis was simulated using the radiation use efficiency method, while hydrology and soil evaporation were simulated using the Ritchie Water Balance and Suleiman-Ritchie methods respectively. Phosphorus and Potassium were not simulated, while Nitrogen was added according to experimental conditions.

### 2.4 CERES maize model evaluation

The 2015 field experiments were used for model calibration. Rainy and dry season data from BUK, SMR and LER were used for model calibration, while rainy and dry season data from DBT were used for model evaluation. The three locations selected for calibration were optimum sites and thereby deliberately selected for calibrating the model, while the last location was not optimal and thereby deliberately selected for model evaluation. The model was calibrated and evaluated following the procedures of Adnan et al. (2019). Genotype specific parameters (GSPs) for the 16 maize varieties were calibrated and evaluated previously by Adnan et al. (2019) and were used in this study (Table 2). The cultivar coefficients were fixed in the calibration and validation exercise, while soil and weather of the different environments were inputted. The records of soil and weather data for individual locations that were already initialized into the *SBuild* and *Weatherman* utilities were used for calibrations. The actual dates of planting, fertilizer application, and harvest were also inputted into the model.

**Table 2:** Calibrated genotype specific parameters of the 16 maize varieties used in the study.

Variety	P1*	P2	P5	G2	G3	PHINT
	(^o^C days)	(days)	(^o^ C days)	Kernel plant^−1^	(mg day^−1^)	(^o^C day tip^−1^)
Sammaz 54	227.4	0.01	518.3	523.3	6.91	42.10
Sammaz 28	192.3	0.01	527.8	514.3	6.99	36.90
Ife Hybrid 5	213.7	0.01	511.6	518.7	7.09	40.00
Ife Hybrid 6	223.6	0.01	520.7	606.7	7.47	35.70
Early White	270.0	0.01	614.3	713.4	6.58	45.00
Sammaz 32	282.0	0.01	601.0	822.0	6.55	45.04
Sammaz 34	287.0	0.01	596.0	827.0	6.77	40.00
Sammaz 41	283.6	0.01	550.7	806.9	7.76	37.00
M1026−10	288.1	0.01	683.4	819.3	7.80	45.50
M1227−12	288.6	0.01	679.2	816.4	7.72	45.50
IWDC2	290.2	0.01	692.7	829.6	8.51	42.90
M0926−8	289.8	0.01	781.8	834.1	8.42	41.20
Oba Super 9	293.1	0.01	768.1	828.7	7.83	45.00
Sammaz 11	298.6	0.01	772.9	830.7	7.80	45.00
TZL-COMP4	293.7	0.01	769.2	786.7	7.59	39.98
TZBSR	294.1	0.01	789.3	846.9	7.17	45.00

P2 = Delay in development for each hour that day-length is above 12.5 h.

P5 = Thermal time from silking to time of physiological maturity.

G2 = Maximum kernel number per plant.

G3 = Kernel growth rate during linear grain filling stage under optimum conditions.

PHINT = Thermal time between successive leaf tip appearances.

* P1 = Thermal time from seedling emergence to the end of juvenile phase.

Statistics used for model evaluation includes index of agreement (d-index), root means square error (RMSE), and Nash-Sutcliffe model efficiency (ME). The d-statistic (Eq. 2) was used because it gives a single index of model performance, which covers bias and variability; it also indicates 1:1 prediction better than R^2^ (Willmott and Willmott, 1982). The ME is the most common method of assessing the power of simulation models (Nash and Sutcliffe, 1970). ME (Eq. 3) have values ranging from -∞ to 1, with a value of 1 indicating perfect prediction, a ME value of 0 indicates that model predictions are as accurate as observed mean, while negative ME values indicate that the observed mean is a better predictor than the model (Ritter and Muñoz-Carpena, 2013). RMSE (Eq. 4) varies with growth over time as the magnitude of the growth variables increase, it takes the unit of the variable and an RMSE value that is below 10 % of the mean of compared values is desirable (Hyndman and Koehler, 2006). RMSE was used because it provides a good estimate of comparisons between observed and simulated single measured data (Loague and Green, 1991).

(2) $$d=1-\;\frac{\sum_{i=1}^{n}{(m}_{i}-S_{i})2}{\sum_{i=1}^{n}{(|S}_{\overset{´}{i}}|+{|m}_{\overset{`}{i}}|)2}$$

(3) $$ME=\;1-\;\frac{\sum_{i=1}^{n}{(m}_{i}-S_{i})2}{\sum_{i=1}^{n}{(m}_{i}-\bar{m})2}$$

(4) $$RMSE=\sqrt{\frac{\sum_{i=1}^{n}{{(m}_{i}-S_{i\;})}^{2}}{n}}$$

Where *n* is the number of observations, $S_{i}$ is the simulated data, $m_{i}$ is the measured data, and $\bar{m}$ is the mean of the measured data.

### 2.5 Seasonal analysis (model application)

**Fig. 1:**
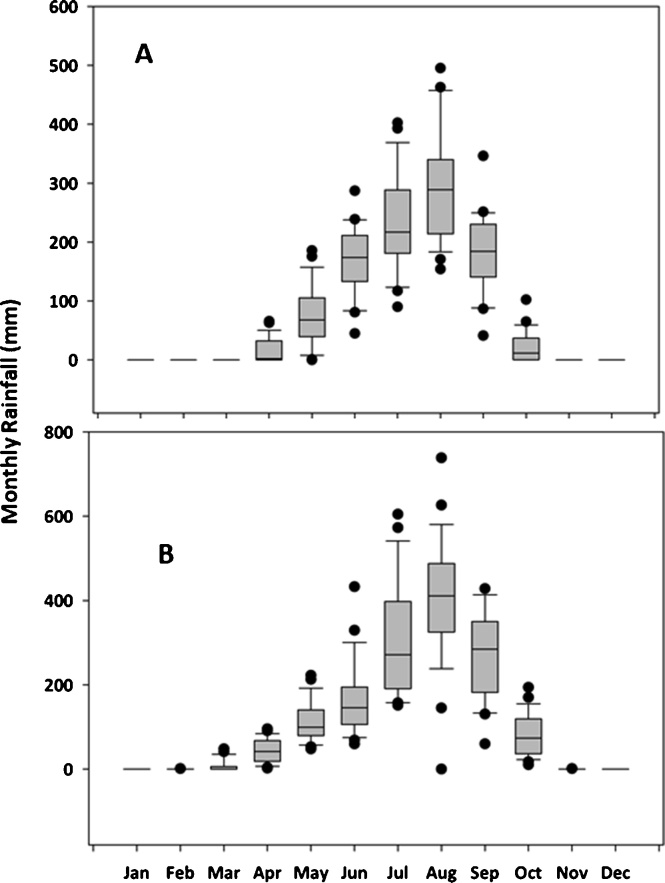
Boxplots showing variation of monthly rainfall over 26 years (1992-2017) for dry savannas (Kano, A) and wet savannas (Zaria, B).

The seasonal analysis tool of DSSAT 4.7 was used to conduct long term sensitivity analysis of the response of the 16 maize varieties in the wet and dry savannas of northern Nigeria. The seasonal analysis was conducted only for the rainy seasons without simulating supplementary irrigation. Long term daily weather data (1992–2017) for Kano (representing dry savanna) and Zaria (representing wet savanna) was collected from the Nigerian Meteorological Agency (NIMET). Box plots showing the rainfall data are depicted in Fig. 1. In the seasonal analysis tool, the model was set to plant automatically when moisture is at optimal and set to harvest at full harvest maturity each year. Optimum recommended nitrogen fertilizer rates (120 Kg N ha^−1^) were applied in two splits, half at planting and the remaining half at 2 weeks after planting (considering moisture availability), both phosphorus and potassium were set at optimum and not simulated.

The weather records indicated that the dry savannas had a shorter growing season with a mean rainfall of 825 mm and a growing season of 3.5 months. The average rainfall in the wet savannas is 1125 mm with a growing period of 5 months. The rains establish earlier in the wet savannas and end later with better distribution than in the dry savannas where rainfall establishes late and ceases early with more than 50 % of the rain received in July and August in most years. Cumulative frequency plots were used to present the results of simulated yields over 26 years.

### 2.6 Estimating GEI and stability analysis

To evaluate the potential of using simulated data in determining the magnitude of GEI and stability of maize varieties, data from separate experiments conducted across all four locations and two seasons in 2016 were used. Each location and season combinations were considered as a unique environment giving a total of eight environments (Table 1). Among the 16 maize varieties used in the present study, four varieties were early, four were extra early, four were intermediate and four were late maturing. Simulations were done separately for the two profile pits in each location to get simulated grain yield with two replications. Observed grain yield data from detailed experiments and simulated grain yields from the calibrated model were subjected to analysis of variance using JMP version 14 software (SAS, 2018). After testing for variance homogeneity, a combined analysis of variance was performed to separate the total variation into components due to genotype/variety (G), environment (E) and genotype × environment interaction (GEI) effects. Genotype was treated as a fixed effect while replication, environment and GEI were regarded as random effects.

Because GEI was found to be statistically significant, additional statistics were calculated to determine the stability of each genotype over the eight environments for both observed and simulated data. To adequately evaluate the potential of using simulated data in determining stability, different stability models were used. Univariate stability models based on regression and variance estimates were first considered. According to the regression model, stability is measured based on mean grain yield, the slope of the regression line (*bi*) and the sum of squares for deviation from regression (*S^2^d*). A variety with a *bi* value that is not significantly different from unity indicates that the variety is adapted to all environments. A variety with a *bi* value greater than unity indicates a higher sensitivity to environmental change meaning the variety has below average stability and is more responsive to higher-yielding environments. A variety with *bi* values less than unity indicates a measure of greater resistance to environmental change, meaning the variety has above-average stability and therefore more responsive and adaptable to low yielding environments (Dia et al., 2017). Other stability parameters were calculated including three multivariate parametric and one non-parametric stability measures. The parametric measures include Wricke’s stability ecovalence (Wricke, 1966), Shukla’s stability variance (Shukla, 1972), and an index that uses both stability variance and ecovalence (SIGMA) (Kang et al., 1987). The Kang yield stability index (Kang YSi) (Kang, 1993) was the non-parametric index adopted, it considers both mean yield and stability variance. Also, AMMI stability value (ASV) was calculated following methods described by Purchase et al. (2000). Varieties with the lowest values were considered to be the most stable for comparisons using ecovalence, stability variance, ASV and SIGMA, (Temesgen et al., 2015). For Kang YSi however, only varieties with stability values greater than the mean stability are considered stable. All the stability parameters (except ASV) were estimated using the R-software through an R-language program (RG × E) developed by Dia et al. (2017). The corrected Akaike Information Criterion (AICc) was used to select the best fitting stability model. The smaller the AICc value, the better the model performance, and the varietal ranking of the selected model was given the highest relevance.

## 3 Results

### 3.1 Model calibration and evaluation

**Fig. 2:**
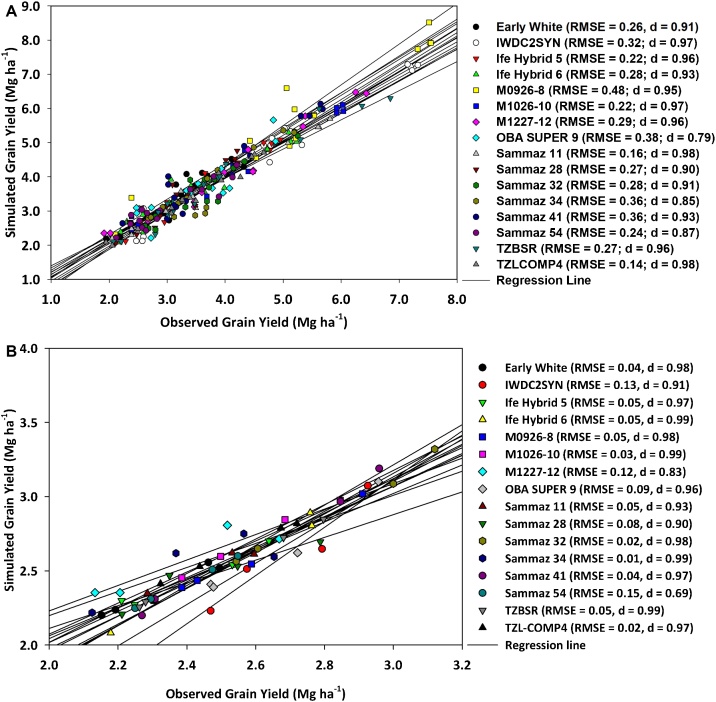
A: Agreements between observed and simulated grain yields of the 16 maize varieties for the model calibration. RMSE = Root Mean Square Error (Mg ha^−1^), d = index of agreement. B: Agreements between observed and simulated grain yields of the 16 maize varieties for the model evaluation. RMSE = Root Mean Square Error (Mg ha^−1^), d = index of agreement.

The result of the model calibration of grain yield for the 16 maize varieties across three locations is shown in Fig. 2 A. All the varieties had RMSE values that were less than 10 % of the mean and d-index values > 0.8 except for OBA SUPER 9 with a value of 0.79. For model evaluation using a separate environment (Fig. 2B), there were good agreements between observed and simulated grain yields as shown by high model statistics. All the varieties recorded d-index values > 0.7 except for Sammaz 54. For the calibration dataset, the model was less efficient in simulating the number of days to anthesis (DTA). One extra early, two early, one intermediate and one late variety had model efficiency values below 0.5 (Table 3). Generally, there was an over-estimation of the DTA for all the early varieties, while for extra early and intermediate varieties only one variety each was over-estimated. For the model evaluation data set, a similar trend was observed, although, the overall model calibration and evaluation statistics were all within acceptable ranges for all the varieties (Table 3). The model was more efficient in estimating biomass at harvest as shown by small RMSE (0.16 – 0.84), lower average bias (0.17 – 0.61 Mg ha^−1^) and high ME that are close to 1 (0.41 – 0.85) (Table 4).

**Table 3:** Result of calibration and evaluation of number of days to anthesis for 16 maize varieties across multiple locations.

Varieties	Calibration dataset				Evaluation dataset			
	Obs	bias	ME	RMSE	Obs	bias	ME	RMSE
*Extra Early*								
Sammaz 54	49	−0.2	0.79	0.54	50	0.4	0.58	0.67
Sammaz 28	48	0.3	0.41	0.58	49	0.3	0.49	0.66
Ife Hybrid 5	50	0.4	0.60	0.77	50	0.2	0.32	0.83
Ife Hybrid 6	48	0.2	0.66	1.07	50	−0.3	0.52	1.12

*Early Varieties*								
Early White	52	−0.8	0.51	1.01	53	0.9	0.52	0.88
Sammaz 32	53	−0.3	0.47	0.88	53	0.2	0.51	0.73
Sammaz 34	52	−0.2	0.79	0.80	53	0.0	0.67	0.76
Sammaz 41	53	−0.4	0.46	0.79	54	0.6	0.43	0.81

*Intermediate*								
M1026−10	56	0.2	0.84	0.99	57	−0.4	0.66	1.01
M1227−12	55	0.3	0.67	0.52	56	−0.6	0.53	0.69
IWDC2	57	0.2	0.50	0.38	57	−0.3	0.47	1.02
M0926−8	58	−0.4	0.42	0.90	57	−0.5	0.51	0.78

*Late*								
Oba Super 9	60	0.4	0.89	0.77	60	0.0	0.73	0.84
Sammaz 11	61	0.2	0.57	1.22	60	0.0	0.49	0.98
TZL-COMP4	62	0.0	0.65	0.56	61	0.2	0.52	0.62
TZBSR	61	0.2	0.44	0.73	61	0.0	0.43	1.01

**Table 4:** Result of calibration and evaluation of biomass yield at anthesis for 16 maize varieties across multiple locations.

Varieties	Calibration dataset				Evaluation dataset			
	Obs	bias	ME	RMSE	Obs	bias	ME	RMSE
*Extra Early*								
Sammaz 54	6.55	0.49	0.67	0.28	6.19	0.62	0.59	0.39
Sammaz 28	6.88	0.35	0.79	0.34	6.73	0.47	0.62	0.42
Ife Hybrid 5	7.30	0.44	0.57	0.49	7.41	0.61	0.49	0.52
Ife Hybrid 6	6.92	0.51	0.51	0.29	7.16	0.73	0.53	0.22

*Early Varieties*								
Early White	6.99	0.37	0.85	0.37	6.59	0.19	0.73	0.41
Sammaz 32	7.62	0.18	0.64	0.45	8.11	0.22	0.67	0.39
Sammaz 34	7.68	0.61	0.80	0.54	8.02	0.37	0.72	0.57
Sammaz 41	8.83	0.44	0.66	0.75	8.93	0.21	0.58	0.67

*Intermediate*								
M1026−10	8.48	0.38	0.84	0.34	9.03	0.28	0.76	0.28
M1227−12	8.56	0.44	0.67	0.36	8.96	0.37	0.52	0.19
IWDC2	9.13	0.24	0.50	0.37	9.19	0.33	0.42	0.47
M0926−8	11.17	0.43	0.42	0.84	11.31	0.28	0.39	0.92

*Late*								
Oba Super 9	8.61	0.33	0.41	0.16	8.31	0.41	−0.51	0.37
Sammaz 11	9.73	0.17	0.57	0.36	8.97	0.28	0.36	0.49
TZL-COMP4	8.41	0.17	0.58	0.20	8.32	0.32	0.49	0.37
TZBSR	10.08	0.63	0.80	0.36	9.19	0.31	0.61	0.32

### 3.2 Observed and simulated grain yields

The effects of variety, environment and the GEI were highly significant (P ≤ 0.001) for both observed and simulated grain yields (Table 5). The environmental effect explained 67 % of the total variance for the observed grain yield and 64 % for simulated grain yield. The main effect of variety explained 19 % of the observed variation and 21 % of the simulated variation for grain yield. The GEI effect explained 13 % and 15 % of the observed variation in observed and simulated grain yields, respectively. This result shows that the variance components of observed and simulated yields are very similar and the variance component of GEI is considerable when compared to the variance component of the variety.

**Table 5:** ANOVA results with variance components for observed (Obs) and Simulated (Sim) grain yields of 16 maize varieties across 8 environments.

Source	Sum of Squares		Mean Squares		% Variance estimate	
	Obs	Sim	Obs	Sim	Obs	Sim
Variety	75.41	85.57	5.03***	5.70***	19.2	20.6
Environment	261.73	265.27	37.39***	37.90***	66.7	64.0
Variety*Environment	50.89	63.49	0.48***	0.60***	13.0	15.3
Rep	0.02	0.27	0.02^ns^	0.27^ns^	0.0	0.1
Error	4.58	0.08	0.04	0.00	1.2	0.0
Total	392.63	414.68			100	100

*** Significant at the 0.001 probability level, ns = non-significant.

**Fig. 3:**
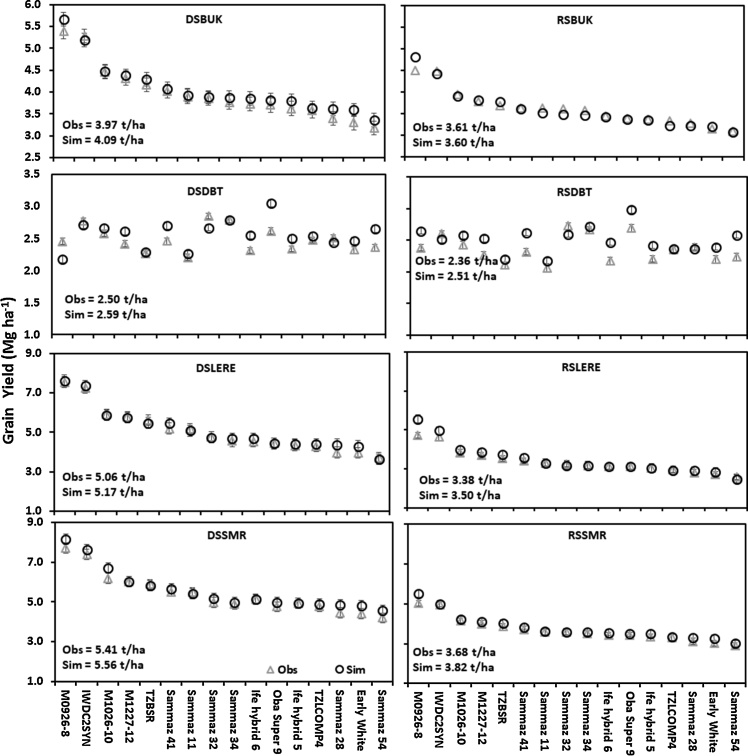
Observed and simulated grain yields of different varieties across locations in the rainy and dry seasons of 2016. V1 = Early WhiteV9 = M0926−8 V2 = Sammaz 41V10 = Oba Super 9 V3 = Sammaz 28V11 = TZBSR V4 = M1026−10V12 = Ife Hybrid 5 V5 = M1227−12V13 = Ife Hybrid 6 V6 = Sammaz 32V14 = Sammaz 54 V7 = Sammaz 34V15 = Sammaz 11 V8 = IWDC2V16 = TZL−COMP4

The average observed and simulated grain yields of the varieties ranged from 2.36 to 2.51 Mg ha^−1^ in RSDBT and from 5.41 to 5.56 Mg ha^−1^ in DSSMR (Fig. 3). Among the varieties, M-0926 produced the highest observed and simulated grain yields in all locations except at DSDBT and RSDBT where the highest observed grain yields were recorded for Sammaz 32 and the highest simulated grain yield was recorded for OBA Super 9. Sammaz 54 and Early white variety produced the lowest grain yields in all the environments except in DSDBT and RSDBT where the two varieties produced higher yields than M-0926. Yields were higher in the dry season environments than in the rainy season across all locations, while the simulated yields were higher than observed yields in 97 % of the data presented. The highest yielding varieties produced consistently highest grain yields across all environments for both observed and simulated grain yields except in the non-optimal environments in Dambatta rainy and dry seasons.

### 3.3 Stability analysis

The best fitting models based on the lowest AICc value for both observed and simulated grain yield (Table 6) were the slope of regression (372.7 for observed and 381.6 for simulated) and the ASV model (392.3 for observed and 394.7 for simulated). The parameters of all the stability models are presented in Table 7. Based on *bi*, the most stable variety using both observed and simulated grain yield was Sammaz 11 (slope = 1.06 for observed and 0.84 for simulated), while the least stable variety was IWDC2 (slope = 3.51 for observed and 3.45 for simulated). Varietal rankings were different for the multivariate parametric models (ASV, Ecovalence, and SIGMA) when both observed and simulated grain yields were considered. For all the three multivariate parametric models, Ife hybrid 6 (ASV = 0.57 and 0.69; Ecovalence = 687.2 and 932.9, SIGMA = 77.8 and 109.1) was the most stable variety for both observed and simulated grain yields, while the least stable variety was M0926−8. Generally, lower ASV, Ecovalence and SIGMA values were recorded for the simulated grain yields than for the observed grain yields across all the varieties. Ranking of the varieties was different for observed and simulated grain yields according to Shukla. The most stable variety was Sammaz 28 for both observed and simulated yields, while the least stable variety was Sammaz 41 for observed yields and M0926−8 for simulated yields. Varietal stability ranking according to Kang YSi identified nine stable varieties for observed grain yields and eight stable varieties for simulated grain yield. Ife hybrid 6 and Sammaz 32 have the highest stability ranking for observed grain yields according to Kang YSi, while the highest-ranking variety for simulated grain yield was Ife hybrid 6. The lowest ranking variety according to Kang YSi was M0926−8 for observed grain yield, while M1026−10 was the lowest ranking variety for simulated grain yields.

**Table 6:** Corrected Akaike Information Criterion for the parametric stability models.

Model	Observed	Simulated
Slope (*bi*)	372.7	381.6
ASV	392.3	394.7
Ecovalence	398.6	404.3
SIGMA	617.2	609.8
Shukla	401.6	411.9

**Table 7:** Mean grain yield and stability parameters for slope of regression, ASV, Ecovalence, SIGMA, Shukla and Kang YSi for observed and simulated grain yields of 16 maize varieties across the environments.

Genotype	GY*		Slope (*bi*)		ASV		Ecovalence			SIGMA		Shukla				Kang YSi	
	Obs	Sim	Obs	Sim	Obs	Sim	Obs	Sim	Obs		Sim			Obs	Sim	Obs	Sim
Sammaz 54	3.0	3.1	0.20	0.27	3.17	2.71	3612.3	6001.5	555.1		936.6			176.1	277.7	−10	−10
Sammaz 28	3.2	3.4	1.62	1.64	2.82	1.04	3034.1	1021.8	460.8		123.6			**70.6^a^**	**54.6^a^**	−8	1
Ife Hybrid 5	3.4	3.6	1.92	0.71	0.98	0.72	839.9	2129.8	102.5		304.5			120.2	362.3	−5	5^+^
Ife Hybrid 6	3.5	3.6	−0.4	1.23	**0.57^a^**	**0.69^a^**	**687.2^a^**	**932.9^a^**	**77.8^a^**		**109.1^a^**			116.7	124.4	**1^a+^**	**4^a+^**
Early White	3.1	3.4	1.2	1.53	2.31	1.15	2496.1	2419.7	372.9		351.9			111.7	173.2	−9	−9
Sammaz 32	3.7	3.7	2.8	2.98	2.23	0.9	1881.0	1156.2	272.5		145.6			110.9	71.7	**1^a+^**	7^+^
Sammaz 34	3.6	3.6	−0.55	−0.54	2.36	1.68	2019.9	2964.9	295.2		440.9			92.7	352.5	−2	−4
Sammaz 41	3.8	4.1	−0.12	−0.63	0.97	0.98	2658.8	3665.1	399.5		555.2			**483.6^b^**	639.2	4^+^	6^+^
M1026−10	4.2	4.2	0.15	0.23	1.96	1.04	1559.7	1699.9	220.2		234.4			150.1	247.1	9^+^	**13^b+^**
M1227−12	4.0	4.1	2.69	2.74	2.01	1.03	2259.1	1706.3	334.2		235.4			156.6	104.8	8^+^	11^+^
IWDC2	4.9	4.9	**3.51^b^**	**3.45^b^**	4.89	3.59	7240.0	9775.2	1147.4		1552.8			168.6	526.9	10^+^	10^+^
M0926−8	5.0	5.4	2.63	3.09	**6.41^b^**	**4.73^b^**	**12593.9^b^**	**17327.9^b^**	**2021.5^b^**		**2785.9^b^**			474.6	**1139.5^b^**	**11^b^**^+^	11^+^
Oba Super 9	3.5	3.7	1.63	1.71	2.75	1.93	3083.2	5999.5	468.8		936.3			161.5	593.2	−3	1
Sammaz 11	3.6	3.7	**1.06^a^**	**0.94^a^**	0.78	0.79	1405.9	1223.2	194.9		156.5			171.9	187.9	6^+^	5**^+^**
TZL-COMP4	3.4	3.4	2.04	1.83	1.68	0.71	2349.4	3102.7	348.9		463.4			253.6	367.9	−7	−8
TZBSR	3.9	3.8	0.14	0.23	2.16	1.18	3170.5	2359.8	483.0		342.0		319.1		179.9	6^+^	2

* GY = Grain Yield averaged across environments (Mg ha^−1^). Boldened entries with parenthesis indicate most stable variety (a) and least stable variety (b) across all environments. Varieties having a cross as superscript are the only stable varieties according to Kang YSi.

### 3.4 Long-term varietal simulations

The maximum, minimum and mean simulated grain yields for 26 years in the wet and dry Savannas using the seasonal analysis tool of DSSAT version 4.6 is shown in Table 8. Varieties IWDC2 and M0926−8 produced maximum yields that are > 5 Mg ha^−1^ in the dry savanna and > 7.5 Mg ha^-1^ in the wet savanna. For some varieties, minimum yields were below 3 Mg ha^−1^ in the dry savanna and above 4.5 Mg in the wet savanna. The highest mean grain yield in the dry savanna was simulated for IWDC2, while in the wet savanna the highest yield was recorded for M0926−8. Sammaz 54 recorded the lowest mean grain yield in the dry and wet savannas. All the late-maturing varieties (OBA SUPER 9, TZBSR, Sammaz 11, and TZLCOMP4) recorded mean grain yields below 3 Mg ha^−1^ in the dry savannas and above 5.5 Mg ha^−1^ in the wet savannas. A 58 % mean yield difference was observed between dry and wet savannas for Sammaz 11, while for Sammaz 54 a mean yield difference of only 8% was observed between the dry and wet savannas. For the highest yielding variety (M0926−8), a yield increase of 39.5 % was observed when planting was done in the dry savannas compared to that of the wet season.

**Table 8:** Maximum, minimum, and mean grain yields for 26-year seasonal analysis of 16 maize varieties using CERES-Maize model.

Varieties	Dry Savanna				Wet Savanna			
	Max.	Min.	Mean	Std. Dev	Max.	Min.	Mean	Std. Dev
Sammaz 54	3.8	1.4	2.3	0.47	3.6	1.0	2.5	0.69
Sammaz 28	3.6	0.9	2.6	0.66	3.8	1.8	2.6	0.77
Ife Hybrid 5	3.6	1.1	2.4	0.72	3.1	1.4	2.5	0.44
Ife Hybrid 6	4.3	1.3	2.8	0.87	4.4	1.6	2.9	0.62
Early White	4.4	2.0	3.1	0.69	5.2	1.0	3.3	1.05
Sammaz 32	4.8	2.1	3.4	0.78	5.1	2.2	3.6	0.91
Sammaz 34	4.4	2.0	3.3	0.79	4.9	2.7	3.8	0.71
Sammaz 41	4.1	2.0	3.2	0.53	5.7	2.9	4.1	0.76
IWDC2	5.1	2.9	4.0	0.94	7.5	4.8	5.8	0.69
M0926−8	5.4	2.8	4.1	1.02	7.8	4.9	6.2	0.87
M1026−10	4.6	2.4	3.4	2.98	7.7	4.8	6.1	0.76
M1227−12	4.7	2.4	3.5	0.53	6.9	4.4	5.5	0.58
OBA SUPER 9	3.5	1.6	2.7	0.78	7.0	3.5	5.6	0.83
TZBSR	3.5	1.8	2.8	0.78	7.7	4.0	6.1	0.85
Sammaz 11	3.3	1.3	2.3	0.47	6.9	3.4	5.5	0.84
TZLCOMP4	3.7	1.4	2.6	0.73	7.3	3.8	5.6	0.90

**Fig. 4:**
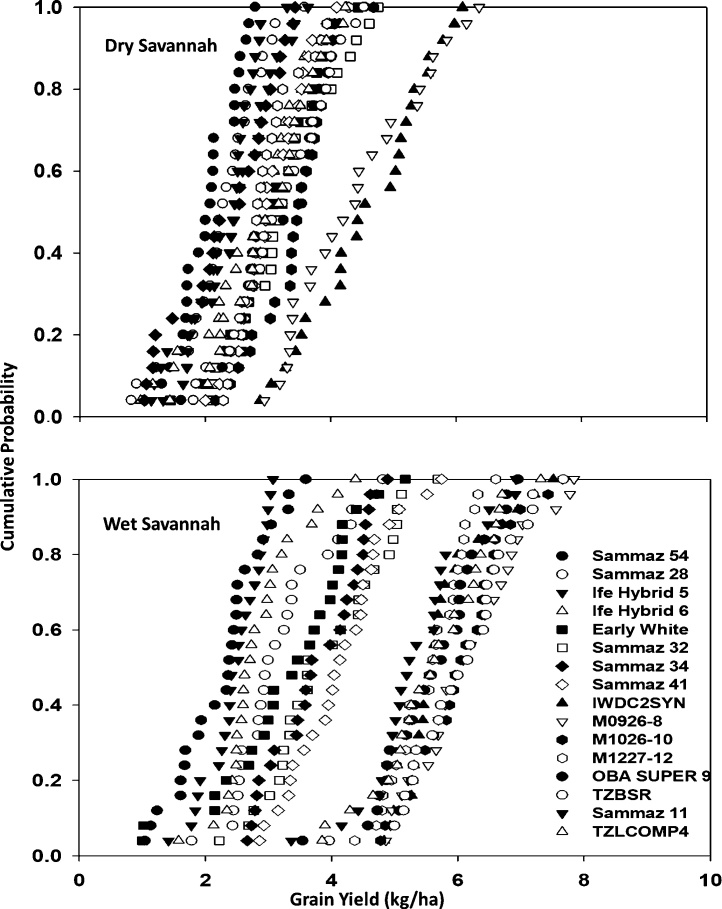
Cumulative probability plot for 26 years seasonal analysis of maize grain yield in the dry and wet savannas.

Fig. 4 shows the cumulative function (CF) plots of 26-years simulated grain yield for the 16 varieties in the dry and wet savannas. In the dry savannas, the highest yielding varieties were IWD C2 and M0926−8. Yields were always below 6 Mg ha^−1^ for all the varieties except the two high yielding varieties where yields exceeding 6 Mg ha^−1^ were simulated in 20 % of the years. The difference in yield among the varieties in the dry savannas was not very high. Both extra early, early and intermediate maturing varieties produced similar grain yields (largely < 4 Mg ha^−1^) in about 75 % of the years simulated. The late maturing varieties produced lower grain yields than the early varieties in all the simulated years and produced equal or more grain yields than the extra-early varieties in only five of the 25 years simulated in the dry savannas. In the wet Savannas, nine out of the 16 varieties produced yields > 5 Mg ha^−1^ in 19 out of the 25 years simulated, while yields below 2 Mg ha^−1^ were recorded for only 6 varieties in 2 out of the 25 years simulated. The intermediate and late varieties produced the highest yields in the wet savannas, with all the intermediate varieties producing yields >5 Mg ha^−1^ in all the years and the late varieties producing yields < 4 Mg ha^−1^ in 18 out of the 25 years simulated.

## 4 Discussion

Selection of appropriate maize varieties that can withstand both biotic and abiotic site-specific problems is one of the major agronomic decisions that could lead to significant maize yield increases in the Nigerian Savannas. The conduct of multi-environmental trials (METs) by breeders and agronomists to assess varietal stability and maximum adaptability to target environments before release has been a long practice (Becker and Leon, 1988; Badu-Apraku et al., 2003, 2012; Seyoum et al., 2019). When properly calibrated and evaluated crop models could complement these METs and provide robust data for improved stability analysis and provide insights into existing genotype by environment interactions.

The outcomes of model calibrations and evaluation from the current study between observed and simulated data show the efficiency and robustness of the model. The accurate prediction of phenology (days to anthesis and days to physiological maturity) were indications that the calculated P1 and P5 values for the varieties used in the *genotype file* were close to the actual values for all the varieties. Accurate prediction of phenology is a major step in the modelling process (Archontoulis et al., 2014), this is because accurate phenology prediction results in the proper estimation of all genotypic variations that affect the leaf area development, biomass production, and grain yield (Robertson et al., 2002). Two environments (DSDBT and RSDBT) were non-optimal environments due to very high temperatures (above 40 °C) during the growing period for DSDBT and the evidence of moisture stress for RSDBT. However, the model was able to reproduce the varietal responses in these environments. The early varieties had short vegetative and reproductive stages while the intermediate and late varieties had relatively longer vegetative and reproductive stages. In the optimal environments, the late and intermediate maturing varieties produced higher grain yields because they took a long time to grow and mature and therefore had longer grain-fill durations. In the stressed environments, the early and extra-early varieties produced higher yields because they took a shorter time to complete vegetative and reproductive developments and escaped most of the stress periods.

Prediction of grain yields for the calibration and evaluation dataset was highly accurate for 15 out of the 16 varieties, with only one variety (Sammaz 54) had values that are slightly above average (Fig. 2). In the CERES-Maize model, grain yield is mostly affected by canopy interception of incident radiation, radiation use efficiency (RUE) and harvest index (Tollenaar and Lee, 2006). Accurate grain yield prediction in a crop modelling exercise is the most important step needed for the improvement of crop management, measuring GEI and varietal stability (Pantazi et al., 2016). Observed and simulated grain yields in each environment were quite variable with two major varieties (M0926−8 and IWDC2) having the highest grain yields in six out of the eight environments. The variation in the environments influenced the final grain yields recorded for all the varieties. Only varieties with tolerance to drought and heat stress were able to produce reasonable yields in poor environments. The rooting parameter which differentiates cultivars due to their ability to tolerate drought and heat stress was calibrated together with the GSPs. This gave the model the ability to capture well, the performance of the drought tolerant varieties in poor locations like Dambatta. In all the environments and for all the varietal groups, the hybrid varieties produced the highest grain yields, however, IWDC2 which is an open pollinated variety (OPV) produced yields similar to the highest yielding hybrid. In the high yielding environments, higher grain yields were recorded in the dry season than in the wet season, this is because of the clear skies which lead to high irradiance and subsequently high RUE (Lindquist et al., 2005). Also, during the dry season, temperatures were optimal for photosynthesis and dry matter allocation. Another reason for the high yields is that during the dry season, all the water requirements were met by irrigation with minimal run-off, while in the rainy season, the high amount of rainfall during July and August could facilitate leaching of a lot of the applied fertilizers thereby affecting growth and yield. The model was able to reproduce these observed anomalies because, in the ecotype files, RUE and canopy light extinction coefficient for daily PAR (KCAN) were adjusted to capture the seasonal variations and effect of supra-optimal temperatures (Lindquist et al., 2005; Zhang et al., 2014). In the specie file, coefficients that represent the effect of temperature on photosynthesis (PRFTC) and relative grain fill duration (RGFIL) were manually adjusted for the tolerant and susceptible varieties to capture the effects of high temperatures on grain yield.

Only a few studies have reported the applicability of crop models in simulating GEI and even fewer have reported using stability analysis techniques in ranking/analyzing model simulated grain yields (Chapman et al., 2003, 2002a; Cooper et al., 2014; Hammer et al., 2006; Salmerόn et al., 2017; Seyoum et al., 2018). Many studies have reported the wide variation of maize producing environments in Nigeria (Badu-Apraku et al., 2015, 2012; Oyekunle et al., 2017). This is the reason why varietal recommendations must be location-specific. In the current study, the environments accounted for more than 60 % of the variations in observed and simulated grain yields, followed by the genotype (variety) and GEI. Seyoum et al. (2019) reported a high influence of management and environment on maize yield and this confounds yield performances thereby minimizing the utility of genotypes in several environments. Thus, the in-depth study of the yield levels, adaptation patterns and stability of both observed and model simulated yields of maize genotypes in multiple environments become imperative. It becomes more important when ranking varieties using simulated grain yields since the models make many assumptions and generalizations.

The results of stability analysis using the slope of regression (*bi*) show an inconsistent ranking of the varieties for observed and simulated grain yields. All the varieties showed *bi* values that were different from unity signifying that they all had an average response to environments, irrespective of the data type. According to Eberhart and Russell (1966) and Becker and Leon (1988), varieties with *bi* values close to unity and high mean grain yields have a good response to changing environments, hence, they are better adapted and more stable across environments. Based on both observed and simulated grain yields, two varieties (Sammaz 11 and Early White) with high mean grain yields and *bi* values close to unity were found to be more stable than all other varieties. Based on the observed grain yield alone, only Oba Super 9 was stable, while based on simulated grain yield alone, Ife Hybrid 5, Ife Hybrid 6 and Sammaz 28 were stable.

For all the three multivariate parametric models, Ife hybrid 6 was the most stable variety based on both observed and simulated grain yields, while the least stable variety was M0926−8. Stability analysis using variance parameter tests did not rank varieties according to high yield, unlike the regression-based stability analysis. This variation between the regression-based stability ranking and the multivariate parametric stability ranking is due to the difference in the methodology for ranking the different varieties. While the regression methodology considers high mean as a precondition for the varietal stability, the multivariate parametric methods do not consider means for calculation of stability (Dia et al., 2017). All the stability models used were consistent in their stability rankings for both observed and simulated yield of the varieties except for Kang Ysi, a non-parametric model that identified different least stable varieties based on the observed and simulated grain yields. This indicates that the simulated data obtained using the CERES-maize model can be used in determining the magnitude of GEI and stability of maize varieties where field data are not available.

In the long-term simulation studies, a high variation in simulated grain yields was observed for the 16 varieties using long term seasonal analysis in the dry and wet savannas. Yields of the early and extra-early varieties were not significantly different between the two savannas. This is because they are early maturing and were able to complete grain filling before the early cessation of rains that is prevalent in the dry savannas. Excessive rains after maize have reached physiological and harvest maturity could lead to significant reductions in harvested grains (Badu-Apraku and Fakorede, 2017). As this is a common occurrence when early varieties are planted in the wet savannas, lower grain yields were expected from the seasonal analysis, but the model did not simulate low yields for early and extra-early varieties in the wet savanna. This is because the model was not able to simulate yield losses due to continuous rainfall after the crop has reached maturity. Yield loss factors like lodging and fungal attack on grains were not captured in the model simulations. The model simulated high grain yields for the intermediate varieties in both dry and wet savannas, although higher yields were produced in the wet savannas than in the dry savannas. The model simulated very low yields for all the late-maturing varieties in the dry savannas because these varieties take a very long time to reach physiological maturity and the period of their active grain filling coincides with the end of the rainy seasons in the dry savanna. The same varieties produced very high grain yields in the wet savannas, indicating that the length of growing season and amount/distribution of rainfall is adequate for proper growth and performance of the late varieties in this zone.

## 5 Conclusion

Crop simulation models are becoming increasingly important tools for explaining the components of GEI that are observed in plant breeding and evaluation trials. Models are used to provide additional environmental indices or ‘virtual’ entries that could be used in providing robust analysis of varietal performance across multiple observed and simulated environments. This is possible when the calibration and model evaluation are robust enough to capture most of the observed varietal performance across multiple environments. Most of the variations observed for both observed and simulated grain yield in the current study were attributed to differences in environments that play a key role in determining crop performance. All the stability models used gave a similar trend for both observed and simulated grain yields and the *bi* model with the lowest AICc value ranked Sammaz 11 as the most stable variety irrespective of the data source. The analysis showed the reliability of simulated data generated using the CERES-maize model in determining the stability of maize varieties. The long-term stability analysis in the dry and wet savannas showed that the late-maturing varieties produce high yields only in seasons where rainfall distribution is long, the intermediate varieties are good in both long and short seasons, while the early and extra-early varieties are more suitable in seasons with short rainfall distribution. Currently, the Intermediate and late varieties are recommended to the wet savannas, while the early and extra-early varieties are recommended for the dry savannas. Findings from our experiments have shown that intermediate varieties could also be planted in the dry savannas in seasons where early rainfall establishment of rainfall was observed, and when seasonal rainfall advice agencies predict long rainy season with good rainfall distribution.

Taken together, the results from the current study have shown that the CERES-Maize model can correctly predict the GEI and stability of maize varieties. Therefore, the model can be used to predict how varieties will behave in locations and seasons where trial data is unavailable and can complement METs with a view of minimizing cost and time expended during such evaluations.

## CRediT authorship contribution statement

**Adnan A.A:** Conceptualization, Methodology, Investigation, Writing - original draft, Formal analysis, Data curation, Visualization. **Diels J.:** Conceptualization, Supervision, Writing - review & editing. **Jibrin J.M.:** Supervision, Writing - review & editing, Funding acquisition. **Kamara A.Y:** Supervision, Writing - review & editing, Funding acquisition. **Shaibu A.S:** Methodology, Investigation, Formal analysis, Data curation, Writing - review & editing. **Craufurd P:** Project administration, Funding acquisition, Writing - review & editing. **Abebe Menkir:** Resources, Writing - review & editing.

## Declaration of Competing Interest

The authors declare that they have no known competing financial interests or personal relationships that could have appeared to influence the work reported in this paper.
